# Isolation and Characterization of Cellulose Microfibers from Colombian Cocoa Pod Husk via Chemical Treatment with Pressure Effects

**DOI:** 10.3390/polym15030664

**Published:** 2023-01-28

**Authors:** Ana Sofia Hozman-Manrique, Andres J. Garcia-Brand, María Hernández-Carrión, Alicia Porras

**Affiliations:** Grupo de Diseño de Productos y Procesos (GDPP), Department of Chemical and Food Engineering, School of Engineering, Universidad de los Andes, CR 1 No. 18A-12, Bogota 111711, Colombia

**Keywords:** agro-industrial wastes, lignocellulosic material, characterization, reinforcement

## Abstract

One of the current challenges is to add value to agro-industrial wastes, and the cocoa industry generates about 10 tons of cocoa pod husks in Colombia for each ton of cocoa beans, which are incinerated and cause environmental damage. This study characterized the Colombian cocoa pod husk (CPH) and to isolate and characterize cellulose microfibers (tCPH) extracted via chemical treatment and pressure. Chemical and physical analyses of CPH were performed, and a pretreatment method for CPH fibers was developed, which is followed by a hydrolysis method involving high pressure in an autoclave machine with an alkaline medium (6% NaOH), and finally, bleaching of the fiber to obtain tCPH. The tCPH cellulose microfibers were also chemically and physically analyzed and characterized by infrared spectroscopy (FTIR), X-ray diffraction (XRD), scanning electron microscopy (SEM), and thermo-gravimetric analysis (TGA). Chemical and physical characterization showed a decrease in lignin content in tCPH. FTIR analysis showed the absence of some peaks in tCPH with respect to the CPH spectrum; XRD results showed an increase in crystallinity for tCPH compared to CPH, due to a higher presence of crystalline cellulose in tCPH. SEM images included a control fiber treated without high pressure (tCPHnpe), and agglomerated fibers were observed, whereas cellulose microfibers with a mean diameter of 10 ± 2.742 μm were observed in tCPH. Finally, with TGA and DTGA it was confirmed that in tCPH, the hemicellulose and lignin were removed more successfully than in the control fiber (tCPHnpe), showing that the treatment with pressure was effective at isolating the cellulose microfibers from cocoa pod husk.

## 1. Introduction

One of the current challenges in industry is to add value to biomass, microorganisms, and agro-industrial wastes. These transformations will prevent them from turning into waste and having negative impacts on the environment [1]. The number of agro-industrial wastes depends on the type of crop and the country in which they are produced. Generally, agricultural wastes occupy soils for some time until they are burned or stored on house roofs, which leads to environmental degradation and the spread of diseases [2]. In the case of Colombia, the processing of products such as coffee, sugarcane, bananas, and palm oil accounts for about 30% of annual agro-industrial waste, which is incinerated or moved to landfills, affecting air quality and people’s health [3].

The importance of lignocellulosic biomass has been studied to use these agro-industrial wastes and reduce the environmental impact. Indeed, it represents the most abundant renewable organic resource present in the soil and is the only source capable of meeting the world’s energy and chemical needs in a renewable way. Its main components are the polymers cellulose (40–50%), hemicellulose (20–30%), and lignin (20–30%), which are interconnected by non-covalent forces and covalent cross-links [4,5,6].

Regarding the components of lignocellulosic biomass, cellulose is a high-molecular-weight polymer that is entangled in a carbohydrate matrix and provides mechanical strength to natural fibers. Aside from being a homopolysaccharide biopolymer formed by glucose molecules, it is the most abundant polymer in terrestrial biomass and has been implemented in the study and synthesis of composite materials and in the generation of energy through the production of bioethanol [7]. Hemicellulose is a low-molecular-weight polysaccharide that can have some degree of branching and can bind strongly to cellulose fibers through hydrogen bonds [8]. Lignin is a biomass-derived phenolic copolymer that has a non-crystalline random network structure, and its function is to protect the plant from chemical and pathogen degradation [9]. 

Cellulose extracted from lignocellulosic biomass can be used as a reinforcement in composite materials due to its high thermal stability, biocompatibility, and high modulus [10,11]; in addition, cellulose isolated from natural fibers is an alternative to synthetic fibers due to its low density, low cost, and wide availability [12]. In addition, cellulose is an abundant polymer in nature, has low abrasiveness, is renewable, and has a low weight [13,14,15]. Some studies have shown that cellulose as a reinforcement in composite materials requires the extraction of individual nanofibers of high crystallinity to reduce the amorphous region [16]. These extraction processes include chemical, enzymatic, and mechanical treatments, and then it is possible to incorporate cellulose as a reinforcement for polymeric matrices [17].

Agro-industrial wastes in the cocoa industry are significant in Colombia, since this industry has grown considerably in the country. Columbia is the fifth largest cocoa producer in the world and the third largest in Latin America [18]. This waste is of great interest worldwide and in Colombia because it is estimated that 10 tons of cocoa pod husks (CPHs) are generated for each ton of dry cocoa beans [19]. The cocoa pod husk (CPH) is composed of four regions: the epicarp, mesocarp, sclerotic section, and endocarp. This is a fibrous material formed by cellulose, hemicellulose, lignin, and pectin; the quantities vary according to the region in which they are produced due to soil characteristics [20]. The CPH constitutes between 52% and 70% of the wet weight of the fruit and has been used as fertilizer, animal feed, and adsorbent material; its pectin is also extracted due to its high content in the husks [21]. Cocoa pod husks in Colombia are incinerated or decomposed on the outskirts of the crops, which allows the proliferation of microorganisms [22]; however, CPH can be used to extract valuable components, such cellulose—again, a natural polysaccharide of high commercial importance. 

The cocoa pod husk (CPH) is between 19% and 48% cellulose [23,24]. To extract this cellulose, it is necessary to remove as much hemicellulose and lignin from the fiber as possible. Hemicellulose is reported to constitute 8.7% to 12.8% of CPHs, and lignin, 14% to 28% [20]. By achieving effective extraction of cellulose from CPH, it is possible to reinforce polymeric matrices, creating bio-composites with good mechanical properties and high biodegradability [25]; moreover, CPH is an abundant waste from which cellulose can be extracted for potential applications, such as packaging [26].

Some studies show the extraction of cellulose from vegetable fibers; in some cases, alkaline hydrolysis and bleaching are performed to extract cellulose fibers [27]. For cocoa pod husks, studies have reported extracting cellulose nanocrystals with an acid hydrolysis procedure with 64% H_2_SO_4_, followed by an ultrasound treatment [28]. Additionally, Jimat et al. reported the isolation of microcrystalline alpha-cellulose by a pretreatment with 2% NaOH, followed by bleaching and hydrolysis with 12% NaOH at 80 °C [29]. Generally, these studies show reductions in the lignin and hemicellulose present in the fiber, and in most cases, hydrolysis procedures are carried out under high concentrations of acids and bases to extract the cellulose. Therefore, there is an opportunity to study other treatments, using lower concentrations and pressure, to separate lignocellulosic wastes, since this could be a method with advantages such as low costs, a low energy requirement, and a lesser environmental impact than conventional hydrolysis [30]. 

Hence, we characterized the Colombian cocoa pod husk (CPH) and isolated and characterized cellulose microfibers (tCPH). Extraction involved a chemical treatment and pressure, so we explored the influence of the pressure on the cleavage of hemicellulose’s glycosidic bonds and hemicellulose–lignin bonds in the hydrolysis process. The extraction of cellulose microfibers was performed by initial treatment of the fiber, followed by alkaline hydrolysis under elevated pressure in an autoclave machine, and finally, bleaching with sodium hypochlorite. The microfibers obtained were characterized by Fourier transform infrared (FTIR) spectroscopy, X-ray diffraction (XRD), scanning electron microscopy (SEM), and thermogravimetric analysis (TGA). Additionally, lignin quantification of the cellulose microfibers (tCPH) was performed, and the results were compared with those for untreated CPH.

## 2. Materials and Methods

### 2.1. Materials

Cocoa pod pusks (CPHs) were collected from the cocoa plantation in the Andean Region in Colombia by Casa Luker S.A. (Caldas, Colombia). Ethanol (99.5%), benzene (99.8%), sodium hydroxide (NaOH, 97%), glacial acetic acid (C_2_H_4_O_2_, 99%), sodium chlorite (NaClO_2_, 80%), sodium hypochlorite solution (NaClO, 4.00–4.99%), and sulfuric acid (H_2_SO_4_, 98%) were purchased from Sigma-Aldrich (St. Louis, MO, USA).

### 2.2. Extraction of Cellulose Microfibers

**Figure 1:**
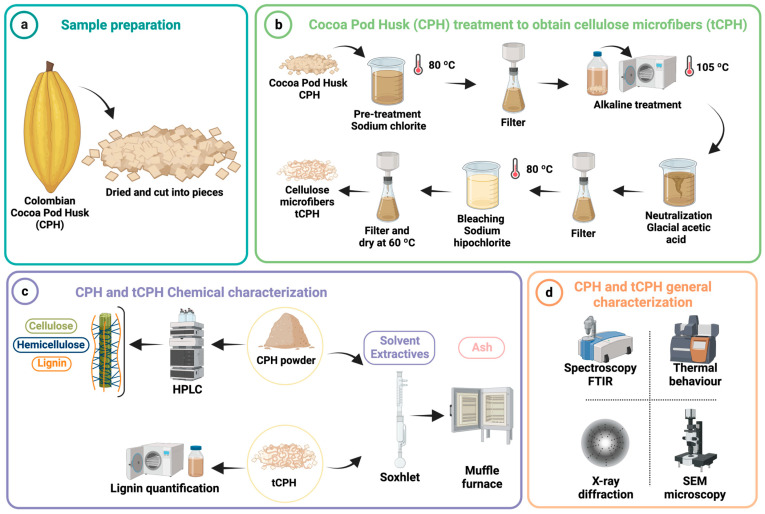
Graphical summary of the procedure to obtain tCPH from CPH and its characterization. (**a**) Sample preparation process. (**b**) Fiber pretreatment, alkaline hydrolysis with high pressure, and bleaching method to obtain tCPH. (**c**) Chemical and physical characterization of CPH and tCPH. (**d**) General characterization of CPH and tCPH. Created with BioRender.com.

Initially, CPHs were cut into small pieces that were heterogeneous in size (maximum 3 cm per side) before the drying process at 105 °C in a forced convection oven (UNB 100, Memmert, Schwabach, Germany) for 12 h to remove water (Figure 1a). For the physical and chemical characterization of CPH, the dried pieces were ground using a knife mill (Universal Cutting Mill PULVERISETTE 19^®^, Fritsch, Idar-Oberstein, Germany). Then, CPH powder was sieved in a shaker (PS 35^®^, PINZUAR, Bogotá, Colombia) with vertical movements at the rate of 60 Hz using a 350 μm mesh.

Figure 1b shows the extraction process of tCPH from CPH. For the extraction of cellulose microfibers (tCPH), CPH fiber was pre-treated with 2% NaClO_2_ for 2 h at 80 °C using a heating plate (Hei-PLATE, Heidolph, Schwabach, Germany) and stirred at 300 rpm with a mechanical stirrer (Hei-TORQUE Ultimate 400, Heidolph, Schwabach, Germany). The resulting fiber was filtered, and an alkaline hydrolysis was performed in an autoclave machine (Multiple Shared Use Autoclave SX-700, Tomy Digital Biology, Tokyo, Japan) at 105 °C for 2 h with 6% NaOH reaching a pressure around 0.12 MPa. After cooling, the sample was filtered again and neutralized with C_2_H_4_O_2_. Finally, it was bleached with 5% NaClO for 2 h at 80 °C using a heating plate (Hei-PLATE, Heidolph, Schwabach, Germany) and stirred at 300 rpm with a mechanical stirrer (Hei-TORQUE Ultimate 400, Heidolph, Schwabach, Germany). The residue (tCPH) was filtered and dried in a convection oven (UNB 100, Memmert, Schwabach, Germany) for 4 h at 105 °C to remove excess moisture.

A control sample (tCPHnpe) was included, and for its extraction, the CPH was pretreated following the same procedure as for tCPH. The hydrolysis procedure was performed at 105 °C for 2 h with 6% NaOH but was carried out using a heating plate (Hei-PLATE, Heidolph, Schwabach, Germany), without pressure. Bleaching of the resulting fiber was performed according to the same procedure as for tCPH.

### 2.3. Chemical Characterization

Figure 1c shows the experimental procedure to perform the chemical characterization of CPH and tCPH. The detailed procedures are presented in the following sections.

#### 2.3.1. Lignocellulosic Content

Cellulose, hemicellulose, and lignin content were measured following the standard procedure (with a NREL TP-510-42618) used for the determination of structural carbohydrates and lignin in biomass. The analysis was performed in triplicate.

The cellulose and hemicellulose contents of CPH fiber were obtained by high-performance liquid chromatography (HPLC) using an HPX-87H column (Agilent, Santa Clara, CA, USA) at 85 °C for 30 min per sample, with a flow rate of 0.6 mL/min of the mobile phase (deionized and degassed water) and a refractive index detector. As part of the assay, a separate set of calibration standards was prepared with analytical-grade filtered glucose, xylose, and arabinose (2.5 mg/mL) after hydrolyzing under the conditions described above. 

For lignin determination of CPH and tCPH, samples (three samples of CPH and tCPH) were hydrolyzed using sulfuric acid (H_2_SO_4_ 72% *w*/*w*) for 1 h at 30 °C in a water bath shaker (SHKA 7000, Thermo Fisher Scientific, Waltham, MA, USA) and exposed to an autoclave cycle for sterilization (SX-700 Multi-Purpose Shared-Use Autoclave, Tomy Digital Biology, Tokyo, Japan). The solid phase was then collected in filtration crucibles to calculate the percentage of lignin.

#### 2.3.2. Ash Content

Ash content in the fiber was determined according to ASTM E1755 in a muffle furnace (FB1310 Thermolyne BenchTop Muffle Furnace, Thermo Scientific, Waltham, MA, USA). Ignition was induced in a porcelain crucible by a temperature ramp from room temperature to 250 °C for 30 min, and then to 575 ± 25 °C. The ash percentage was calculated based on the residual weight and the initial dry sample. Five samples of CPH and tCPH were tested.

#### 2.3.3. Extractive Content

The extractives content was measured to quantify the presence of waxes, fats, and surface resins using the TAPPI T204 method. A Soxhlet apparatus was used; CPH and tCPH were exposed through a heating process with a mixture of ethanol and benzene (1:2 *v*/*v*) for 5 h until it reached its boiling point. Three samples of each material, CPH and tCPH, were tested.

The extractive content was calculated according to Equation (1).

(1) $$\%\;\mathrm{Extractives}=\frac{W_{e}{-W}_{b}}{W_{p}}\cdot 100,$$

where W_e_ is the dry weight of the extract, W_b_ is the weight of the blank residue, and W_p_ is the initial dry weight of the natural fiber.

### 2.4. Physical Characterization

#### Moisture Content

Moisture content was measured following the ASTM D1348 standard using method B. CPH and tCPH were dried in an oven (UNB 100, Memmert, Schwabach, Germany) at 150 °C until differences between weights at consecutive times were not significant (less than 0.005 g). The moisture content was calculated from the change in the sample weight before and after the drying process. Five samples of each material, CPH and tCPH, were tested.

### 2.5. Spectral Analysis (FTIR)

To analyze the chemical groups in both CPH and tCPH samples, they were studied using Fourier transform infrared spectroscopy (FTIR). The spectrum was measured with wavelengths between 4000 and 500 cm^−1^ using the Nicolet iS50 FTIR Spectrophotometer^®^ (Thermo Fisher Scientific, Waltham, MA, USA) and 32 scans at room temperature.

### 2.6. X-Ray Diffraction Analysis

X-ray diffraction (XRD) analysis of one sample each of CPH and tCPH was performed on a Rigaku Ultima III X-ray diffractometer (Tokyo, Japan) in the Bragg–Brentano configuration. A Bragg diffraction angle scan between 5° and 50°, using an increment of 1.5°/s and Kα radiation with a copper (Cu) anode of 40 kV and 40 mA, was used for the test. To identify the phase contents of CPH and tCPH, deconvolution of the XRD profiles was performed using Origin Pro software (Northampton, MA, USA). The crystallinity index (Cr I) was determined using Segal equation shown below [31].

(2) $$\%\;\mathrm{Cr}\;I=\frac{I_{200}{-I}_{\mathrm{am}}}{I_{200}}\cdot 100,$$

where I_200_ is the intensity (in arbitrary units) at the peak 2θ ≈ 22° and I_am_ is the intensity at the peak 2θ ≈ 17°.

### 2.7. Scanning Electron Microscopy (SEM) Analysis

Scanning electron microscopy (SEM) was used to visualize the surface morphology of three types of samples: (i) cocoa pod husk (CPH), (ii) a control sample prepared with alkaline hydrolysis and without pressure (tCPHnpe), and (iii) a sample prepared with alkaline hydrolysis and pressure (tCPH), using a JEOL SEM model JSM-6490LV^®^ (Akishima, Tokyo, Japan) at 10kV. Samples were coated with gold at room temperature to reach the necessary conductivity using a Vacuum Desk IV apparatus^®^ (Denton Vacuum, Moorestown, NJ, USA) and fixed with colloidal glue. Likewise, samples were visualized using energy dispersive X-ray spectroscopy (EDS). The diameters of cellulose microfibers obtained from tCPH were measured using Image J analysis software (Madison, WI, USA). Three images and a total of seven microfibers were measured for tCPH, and a control sample treated without pressure (tCPHnpe) was observed for comparison.

### 2.8. Thermo-Gravimetric Analysis (TGA)

To analyze the thermal stability, thermogravimetric analysis (TGA) was used. Tests were carried out using the TA Instruments Q600 thermogravimetric analyzer^®^ (New Castle, DE, USA), according to the ASTM E1131 standard. For the test, 5.6 mg of samples were placed into platinum sample pans and heated from 30 °C to 600 °C at a heating rate of 10 °C /min in a nitrogen atmosphere with a nitrogen flow rate of 10 mL/min. The analysis was performed for CPH, a control sample with alkaline hydrolysis treatment without pressure (tCPHnpe) and tCPH cellulose microfiber. 

## 3. Results and Discussion

### 3.1. CPH and tCPH Chemical Characterization

The chemical characterization of CPH is shown in Table 1. It shows that cellulose made up almost half of the chemical compounds in the CPH fiber (41.43 ± 0.17%), which is consistent with the literature, since cellulose is the basic structural component of plant cell walls [32]. High cellulose content means an increment in the fiber strength [33]; in addition, cellulose is a promising polymer as a reinforcement material because of its mechanical properties, biodegradability, hydrophilicity, and biocompatibility [34].

The percentage of hemicellulose in the CPH fiber was 14.15 ± 0.76%. One of the primary functions of hemicellulose in the cell plants is giving strength and support to the cell wall through the interactions with cellulose and lignin [35]. As hemicellulose surrounds cellulose fibers, it must be removed from the fibers to expose cellulose fibers. Furthermore, without amorphous hemicellulose, the crystallinity of the natural fiber increases [36], which is related to the increased reinforcing capacity of cellulose in a composite material [37].

The second-most-abundant chemical component in the CPH is lignin (33.04 ± 0.66%). Lignin has the essential function of protecting the plant from microbial and chemical degradation due to its molecular architecture [38]. However, when incorporating fiber in composite materials, a high lignin value affects the fiber–matrix compatibility. Therefore, the lignin removal process, known as delignification, uses chemical treatments to solubilize the lignin and expose the cellulose, increasing the compatibility between the fibers and the polymeric matrices [39,40].

The chemical composition of CPH varies depending on the location from which it originates. This is due to different geographical locations having diverse soil, climatic conditions, and different ways of planting and harvesting. CPH from Colombia has a cellulose percentage about 5% higher than CPH from other cocoa-producing countries, such as Brazil (32.30 ± 1.80%), Nigeria (35.00 ± 2.00%), Ghana (26.10 ± 0.22%) and Malaysia (35.40 ± 0.33%) [20, 38, 41]. In addition, another study has compiled data on added-value biomolecules from CPH showing the chemical characterization of the fiber. These values are close to those obtained for Colombian CPH regarding cellulose percentage (around 35% *w/w*) [42]. This means that CPH from this study is a useful source of cellulose that can be extracted to add value to the waste and reinforce composite materials.

Table 2 shows the characterization of lignocellulosic components of CPH and compares it with other natural fibers added to polymeric matrices as reinforcing material.

According to Table 2, the cellulose percentage of CPH is high compared with most natural fibers, which represents an advantage of this agro-industrial waste. However, the percentage of lignin is also high in relation to other fibers; it should be removed using a delignification method. A study showed the extraction of cellulose from coconut coir using an alkaline treatment to form alkali-treated coir-polyester composites [43]. The same is done for sugarcane bagasse, which is treated with NaOH to reduce the lignin and hemicellulose content and to reinforce matrices of polyethylene, polyvinyl alcohol, polypropylene, etc. [44]. For this reason, treating CPH is an effective way to take advantage of its cellulose content.

**Table 2:** Lignocellulosic content of CPH fibers compared to other natural fibers.

Fiber	Chemical Composition (% *w/w*)			
	Cellulose	Hemicellulose	Lignin	Ref.
cocoa pod husk (CPH)	41.43 ± 0.17	14.15 ± 0.76	33.04 ± 0.66	This study
Coconut Coir	21.07	8.50	29.23	[45]
Sugarcane bagasse	26-47	19–33	14-23	[46]
Banana peel	18.74	20.28	16.77	[47]
Corn husk	45.13	31.15	14.32	[48]
Rice husk	32.67	31.68	18.81	[49]
Cocoa Bean Shell	42.23	14.73	22.68	[50]

Since alkaline treatments can reduce the lignin and hemicellulose contents of natural fibers, Table 3 shows results of chemical characterization of cocoa pod husk (CPH) and cellulose (tCPH) microfibers. For the CPH fibers, cellulose and hemicellulose were categorized, and the percentage of each component is indicated. For tCPH, the percentage of holocellulose is shown, which corresponds to cellulose and hemicellulose.

According to the results in Table 3, there was a reduction in the percentage of lignin in tCPH because of the effectiveness of the hydrolysis and bleaching procedure of the cocoa pod husk. The 14% reduction in lignin in tCPH compared to CPH is shown by the increase in the percentage of holocellulose. In addition, the hydrolysis process also involves the removal of hemicellulose, which indicates an increase in the content of exposed cellulose in the treated fiber [51].

The reductions in the percentages of lignin and hemicellulose in tCPH are indicators of hydrolysis’s effectiveness and are important because some studies show that if considerable amounts of lignin and hemicellulose are removed from a fiber, the hydroxyl groups present in the cellulose can form hydrogen bonds. This improves the mechanical properties of the treated fiber [52,53], and in turn, is an advantage if tCPH is to be incorporated as a reinforcement in polymeric matrices.

Isolating cellulose from natural fibers is of great interest due to its applications in composite materials, packaging, paper, etc. [54]. In addition to being abundant in nature and biocompatible, the mechanical properties of cellulose can be compared to those of other materials, such as glass fibers. Cellulose has a great advantage over other materials because it is a low-density fiber (1.58–1.59 g/cm^3^) and has high specific stiffness, which favors its incorporation in composites [55,56].

As mentioned above, alkaline treatments with sodium hydroxide (NaOH) are effective, since they break the bonds between lignin and hemicellulose, increase the surface area, and provide greater accessibility to the cellulose fraction [57,58]. According to Table 3, the percentage of holocellulose is 11.8% higher in tCPH than in CPH. Cellulose is the major component in this percentage, since hydrolysis affects the acetyl group of hemicellulose, removing most of it [59]. The effectiveness of the alkaline hydrolysis process to remove hemicellulose and lignin increases when performed in an autoclave machine, since in addition to the high temperature in hydrolysis, the pressure increases above atmospheric pressure, favoring the disruption of lignin and cell walls [60]. This chemical characterization showed the effectiveness of the alkaline treatment under pressure and showed the difference between untreated cocoa pod husk (CPH) and the treated fiber (tCPH), related to the decrease in the percentage of lignin and increase in the percentage of holocellulose in tCPH. 

The percentages of extractives and ash were not considerably different between CPH and tCPH. The slight decreases in tCPH are related to hydrolysis, which not only removes hemicellulose and lignin, but also removes fats, waxes, and resins.

### 3.2. Physical Characterization of CPH and tCPH

The moisture content of CPH was 6.72 ± 0.76%, and that of tCPH was 5.91 ± 0.002%. The moisture content of natural fibers is related to the mechanical properties of the constituent materials. Studies show that decreasing the moisture content has advantages in composite materials formed by jute and flax fibers, as decreasing the moisture content increased the Young’s modulus [61,62]. 

The moisture content of tCPH was lower than that of CPHs. This difference is explained by the removal of hemicellulose reducing the absorption of moisture by the fiber, and the alkaline treatment to obtain tCPH probably reduced the percentage of hemicellulose with respect to untreated cocoa pod husk (CPH) [63]. In this way, tCPH cellulose microfibers could be used as reinforcements for composite materials, thereby providing materials with lower porosity and good processability [64].

### 3.3. Spectral Analysis (FTIR)

**Figure 2:**
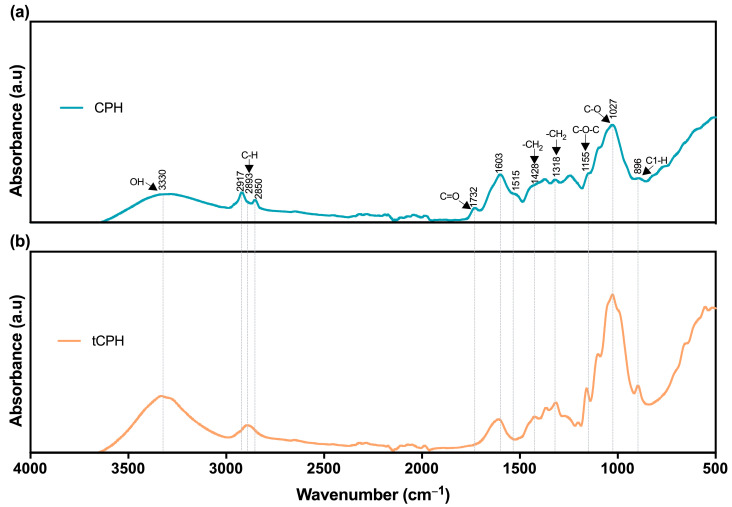
FTIR spectrum of (**a**) CPH fiber and (**b**) tCPH fiber.

The FTIR analysis of CPH is shown in Figure 2a. Through this analysis, the constituents of the raw material were confirmed due to the presence of the main bands of cellulose, hemicellulose, and lignin. Although it is a qualitative analysis, the amplitudes of the peaks could be indicators of the substances’ concentration or the number of molecules with specific vibrations [65]. Therefore, the differences between the amplitudes of the CPH and tCPH spectra (Figure 2b) may be indications of lignin and hemicellulose removal and a higher cellulose concentration in tCPH, supporting the results of the chemical characterization.

First, the peak near 3330 cm^−1^ corresponds to the presence of polysaccharides shown by the hydroxyl groups’ (OH) stretching vibration; additionally, this peak includes inter- and intra-molecular hydrogen bond vibrations in cellulose [66], and it is more pronounced for tCPH, probably due cellulose being at a higher concentration. The next peak located at 2917 cm^−1^ is clearer in the CPH spectrum and corresponds to the stretching in the aliphatic methylene group of fatty acids present in lignin. The same occurred with the peak located at 2850 cm^−1^, corresponding to lignin and hemicellulose asymmetric methyl and methylene stretching; this was observed only for the CPH fiber and means that in the tCPH, the lignin and hemicellulose were largely removed [67,68].

At 2893 cm^−1^ there is a peak only in the tCPH fiber’s spectrum, and this corresponds to C-H stretching in the CH and CH_2_ bonds in cellulose and hemicellulose [69,70]. At around 1732 cm^−1^, there is a peak corresponding to the acetyl and ester groups present in hemicellulose and fiber extracts (C=O bonds) [50,71]; this peak is only observed in CPH’s spectrum, indicating the removal of hemicellulose in tCPH. Additionally, at around 1603 cm^−1^ there are peaks for the aromatic ring of lignin [72], and this peak is present in both CPH and tCPH’s spectra, although it is more pronounced (higher amplitude) in that of CPH, indicating the partial removal of lignin in tCPH due to alkaline hydrolysis. The peak observed near 1428 cm^−1^ corresponds to vibrations of CH and CH_2_ bonds of cellulose [66]. 

A peak at 1318 cm^−1^ is present, more pronounced for tCPH, which corresponds to O-H deformation and CH_2_ rocking vibration in cellulose [73,74]. A pronounced peak in the tCPH spectrum is present at 1155 cm^−1^ and corresponds to cellulose C-O-C stretching [75]. Likewise, the peak at 1027 cm^−1^ corresponds to the C-O stretching in the ring of cellulose [73,75]. Again, in the tCPH spectrum, these peaks are much more pronounced than in that of CPH, indicating a higher concentration of cellulose. Finally, at 896 cm^−1^, a pronounced peak is observed for tCPH, which corresponds to the deformation of the C1-H bond of cellulose [76]. Accordingly, several regions of the tCPH spectrum showed the removal of hemicellulose and part of the lignin. This indicates that there were differences in the chemical composition of the untreated fiber (CPH) and the treated fiber (tCPH), which coincides with the results obtained in the chemical analysis. 

### 3.4. X-ray Diffraction Analysis

**Figure 3:**
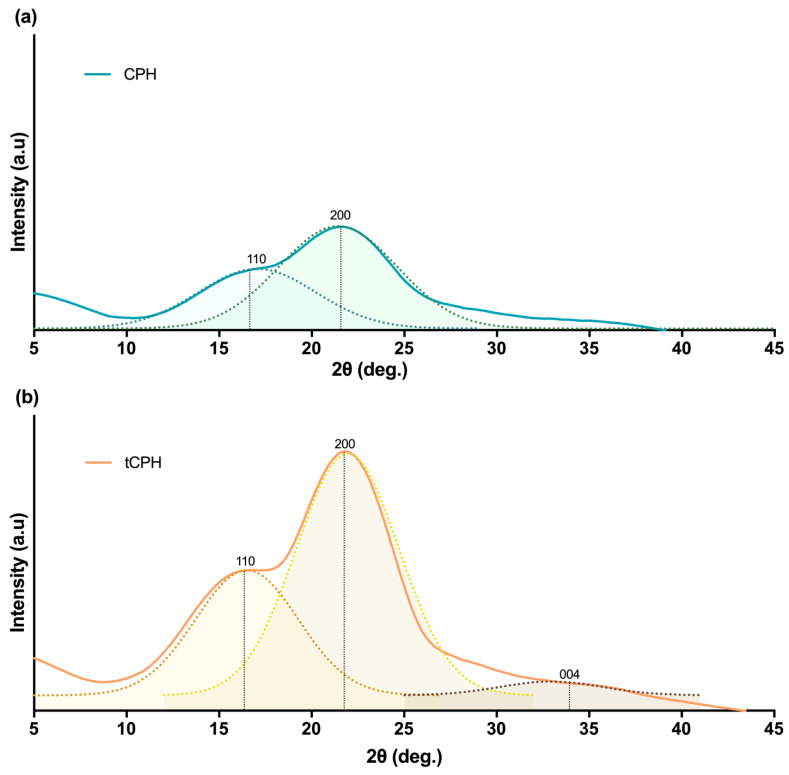
(**a**) XRD pattern for CPH with the deconvolution of main phases. (**b**) XRD pattern for tCPH with the deconvolution of main phases.

X-ray diffraction patterns and deconvolutions for the phases of each pattern for CPH and tCPH fibers are shown in Figure 3. Deconvolution was carried out to identify the differences between the CPH and tCPH patterns, showing that for CPH (Figure 3a), two peaks exist at 2θ values of 16° and 22°. Similar behavior was observed for tCPH (Figure 3b): these peaks were present in the (110) and (200) planes. The common peaks of CPH and tCPH represent cellulose I, and the region between peaks (110) and (200) represents the amorphous components of cellulose [77]. In the case of tCPH, an additional peak can be observed at 34° (Figure 3b), corresponding to the (004) plane of cellulose [78]. 

The greatest difference in the spectra is shown by the fact that for tCPH, the peaks are higher (Figure 3b), which occurred because the amorphous cellulose chains were arranged in the crystalline domain of cellulose, increasing the degree of crystallinity [79,80,81]. The Segal equation allows a good approximation of the degree of crystallinity of CPH or tCPH [31]; the results show that the crystallinity of tCPH was 42.84%, and the crystallinity of CPH was 28.83%. 

The crystallinity index shows that tCPH had a higher proportion of crystalline cellulose than CPH; therefore, alkaline hydrolysis was effective at removing amorphous regions associated with hemicellulose and lignin. In addition, the increased crystallinity of tCPH makes it a good alternative as a reinforcement for composite materials, given its higher resistance to thermal degradation and lower water absorption than natural CPH fiber. The increase in the crystallinity index also means a higher proportion of cellulose crystals in the microfibers obtained from tCPH. Furthermore, it indicates that much of the hemicellulose and lignin in the amorphous region were removed by the alkaline hydrolysis procedure [82]. 

Some studies show that the delignification process increases the crystallinity values of cellulose in vegetable fibers. Increased crystallinity means higher strength and lower water absorption by the fiber, since it is the amorphous regions that absorb the most water [83]. Krishnaiah et al. [84] showed that sisal fiber increases crystallinity when amorphous components are removed by alkaline treatments coupled with ultrasound. Another study showed the extraction of alpha-cellulose and microcellulose from CPH. In that study, a crystallinity index of 43.83% for microcellulose was obtained, which is like that of this study for tCPH (42.84%). However, to extract alpha-cellulose, a hydrolysis with 0.5 M HCl was performed, followed by an additional hydrolysis in triplicate with 1M NaOH, and finally, a hydrolysis with 2.5 N HCl [85]. This was a much longer procedure and required more reagents to obtain results like those of this research, where pressure aided in the removal of lignin and hemicellulose. 

### 3.5. Scanning Electron Microscopy (SEM) Analysis

**Figure 4:**
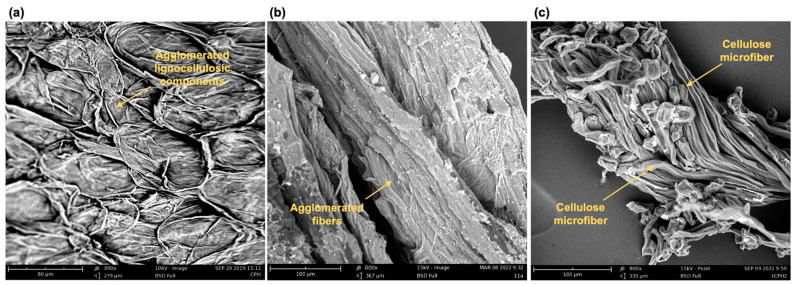
Microscopic structure of (**a**) CPH fiber, (**b**) control sample (tCPHnpe), and (**c**) tCPH cellulose microfibers. All SEM micrographs used 800× magnification.

Figure 4 presents scanning electron microscopy (SEM) images of three types of samples: (i) cocoa pod husk (CPH—Figure 4a), (ii) a control sample prepared with alkaline hydrolysis without pressure (tCPHnpe—Figure 4b), and (iii) a sample prepared with alkaline hydrolysis and a high pressure (tCPH—Figure 4c). Morphological analysis of the longitudinal sections of the CPH (Figure 4a) showed rough sections with fibers attributed to cellulose, hemicellulose, and lignin, randomly arranged and without evident patterns. In tCPHnpe (Figure 4b), agglomerated fibers were observed, and in the tCPH image (Figure 4c), cellulose microfibers are more clearly observable. 

The control sample (tCPHnpe) showed that the treatment without pressure is not completely effective in the removal of hemicellulose, which confirms that it is more effective to add pressure to the alkaline hydrolysis to obtain cellulose microfibers like those of tCPH. Cellulose microfibers from tCPH (Figure 4c) showed an average diameter of 10 ± 2.74 μm; some studies report the extraction of microfibers from other natural fibers, such as coconut, sugarcane bagasse, and sisal. For microfibers obtained from coconut palm, diameters of 10 to 15 μm have been reported, whereas other cellulose microfibers, such as those from sugarcane bagasse, have average diameters of 14 μm, which is close to the diameter of microfibers from tCPH [86,87]. 

Cellulose microfibers have advantages when incorporated into composite materials due to their larger surface area, high strength, and ability to form strong but lightweight materials [88]. The morphology of the tCPH microfibers in the SEM analysis supports what was found in the previous sections. The removal of hemicellulose and most of the lignin after treating cocoa pod husk (CPH) to obtain tCPH resulted in microfibers with homogeneous diameters, with the potential to be incorporated into polymeric matrices for reinforcement.

### 3.6. Thermo-Gravimetric Analysis (TGA)

TGA and DTGA analysis was performed to evaluate the thermal stability of (i) cocoa pod husk (CPH), (ii) a control sample prepared with alkaline hydrolysis without pressure (tCPHnpe), and (iii) a sample prepared with alkaline hydrolysis and high pressure (tCPH), in order to validate what was observed in SEM images. 

**Figure 5:**
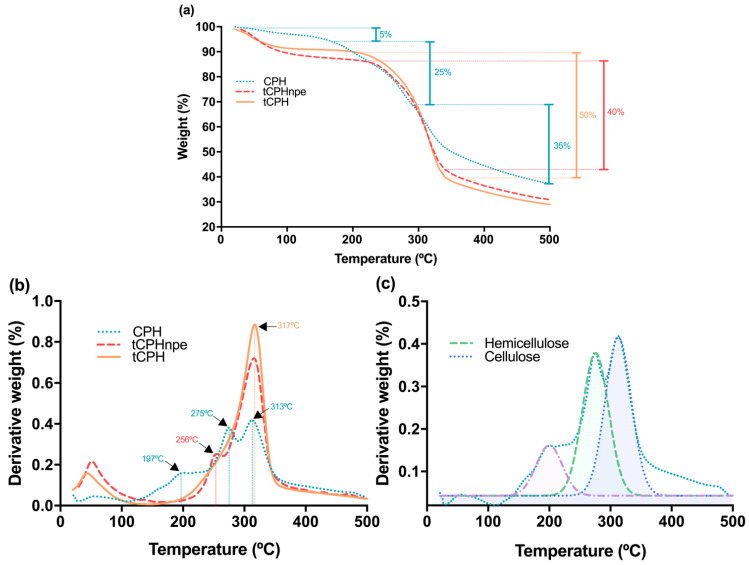
Thermogravimetric curves of CPH, tCPH, and tCPHnpe. (**a**) TGA curves of CPH, tCPH, and tCPHnpe, (**b**) DTGA curves of CPH, tCPH and tCPHnpe, (**c**) DTGA deconvolution for CPH fiber into separate regions of mass loss due to hemicellulose and cellulose decomposition.

According to the TGA results for CPH, tCPHnpe, and tCPH (Figure 5a), the first stage of mass loss of less than 10% occurred between 30 and 100 °C, due to the loss of water from the three fibers [89]. This mass loss has also been described for many lignocellulosic fibers at temperatures between 25 and 150 °C, due to the tendency of water retention in the biomass [90,91,92]. For CPH, 25% of mass loss was because of hemicellulose decomposition, and approximately 35% mass loss corresponded to cellulose and lignin degradation. For tCPH, TGA (Figure 5a) showed a single mass-loss stage of almost 50%, where the degradation of each of the lignocellulosic components cannot be precisely identified, since the pretreatment procedure, alkaline hydrolysis under elevated pressure, and subsequent bleaching of the fiber eliminated large amounts of hemicellulose and lignin. According to the TGA for tCPHnpe, a single mass-loss stage was also observed, but a smaller one than for tCPH, being close to 40%. 

Figure 5b shows the DTGA for CPH, tCPHnpe, and tCPH. It shows more clearly the decomposition stages of hemicellulose, cellulose, and lignin in CPH fibers. CPH is thermally stable until the second decomposition (mass loss of about 35%) located between 200 and 290 °C. This mass loss is due to dehydration, decarboxylation, and decarbonylation of the hemicellulose and some cellulose structures (i.e., cleavage of C-H, C-O, and C-C bonds) [92]. Moreover, in this region, from 200 °C onwards, the deterioration of the aromatic ring of lignin begins [93,94], and pectins present in CPH are also degraded [95]. The third stage of decomposition in CPH occurs near 300 °C, and Figure 5c presents the deconvolution of the DTGA data for CPH to identify more precisely the thermal degradation ranges of hemicellulose and cellulose, coinciding with thermogravimetric analyses present in studies for the same raw material [94,96,97,98,99]. 

In the DTGA for the control sample tCPHnpe (Figure 5b), there is a peak at 256 °C, corresponding to the thermal degradation of hemicellulose and lignin remaining in the fiber [92], and an additional peak at 317 °C, corresponding to cellulose degradation in the fiber. On the contrary, the DTGA for tCPH shows only one stage of decomposition, which occurred between 200 and 400 °C, with a maximum peak at 317 °C. This indicates that higher proportions of hemicellulose and lignin were removed in tCPH than in tCPHnpe, and therefore, the cellulose decomposition stage can be clearly observed.

The difference between the degradation stages of CPH and tCPH according to the DTGA is also worth noting here: the single stage of degradation for tCPH shows that the alkaline treatment under pressure was effective at removing hemicellulose and a large portion of the lignin, which in turn led to an increase in the percentage of cellulose. 

The above is a sign of the effectiveness of the treatment with pressure at the removal of hemicellulose and part of the lignin and agrees with the SEM results, in which cellulose microfibers can be observed for tCPH, whereas agglomerated fibers were present in the control sample and CPH. Finally, thermogravimetric analysis allowed establishing a maximum tCPH treatment temperature of 230 °C, which will prevent thermal degradation.

## 4. Conclusions

The analysis of the chemical composition of cocoa pod husk showed that most of the fiber is composed of cellulose; however, the high percentage of lignin is a limitation for the incorporation of the fibers into polymeric matrices. After isolating the tCPHs’ cellulose microfibers, chemical composition analysis showed a 14% decrease in lignin and an 11.8% increase in holocellulose; spectral analysis showed decreases in lignin and hemicellulose groups in tCPH compared to CPH. These results were also supported by X-ray diffraction (XRD) analysis, which indicated a 14% increase in tCPH crystallinity with respect to CPH, related to an increase in crystalline cellulose in the treated fiber. The results were confirmed by including the control sample tCPHnpe in the SEM analysis, where only in tCPH were cellulose microfibers observed. In addition, the TGA and DTGA showed that in the sample treated without pressure (tCPHnpe), lignin and hemicellulose were not suitably removed, as was observed in tCPH. Therefore, alkaline treatment with pressure was effective at isolating cellulose microfibers from cocoa pod husks, and they can be evaluated for future incorporation into composite materials to add value to the cocoa industry’s waste. 

## Figures and Tables

**Table 1:** Chemical composition of CPH fiber.

Fiber	Chemical Composition (% *w/w*)				
	Cellulose	Hemicellulose	Lignin	Ash Content	Solvent Extractives
CPH	41.43 ± 0.17	14.15 ± 0.76	33.04 ± 0.66	11.30 ± 0.32	3.77 ± 0.001

**Table 3:** Comparison between the chemical compositions of CPH and tCPH.

Fiber	Chemical Composition (% *w/w*)				
	Holocellulose		Lignin	Ash Content	Solvent Extractives
	Cellulose	Hemicellulose			
CPH	41.43 ± 0.17	14.15 ± 0.76	33.04 ± 0.66	11.30 ± 0.32	3.77 ± 0.001
tCPH	67.39 *		19.03 ± 2.90	10.92 ± 0.55	2.66 ± 0.02

* Calculated from the difference in total constituents.

## Data Availability

The data presented in this study are available on request from the corresponding author.
